# Development of Real-Time and Lateral Flow Dipstick Recombinase Polymerase Amplification Assays for the Rapid Field Diagnosis of MGF-505R Gene-Deleted Mutants of African Swine Fever Virus

**DOI:** 10.3390/vetsci12030193

**Published:** 2025-02-20

**Authors:** Jizhou Lv, Junhua Deng, Yu Lin, Dongjie Chen, Xiangfen Yuan, Fang Wei, Caixia Wang, Xiaolin Xu, Shaoqiang Wu

**Affiliations:** 1 Institute of Animal Quarantine, Chinese Academy of Inspection and Quarantine, Beijing 100176, China; lvjz@caiq.org.cn (J.L.); linyu.work@foxmail.com (Y.L.);; 2 Center for Biosafety, Chinese Academy of Inspection and Quarantine, Sanya 572024, China; 3 Technology Innovation Center of Animal and Plant Product Quality, Safety and Control, State Administration for Market Regulation, Beijing 100176, China

**Keywords:** African swine fever, MGF-505R gene, B646L gene, recombinase polymerase amplification (RPA)

## Abstract

Pigs are susceptible to the deadly infectious disease known as African swine fever (ASF), which is brought on by the African swine fever virus (ASFV). As such, prompt and precise disease detection is essential. Deletion of the virulence-related genes MGF-505/360 and EP402R generated from the virulent genotype II virus significantly reduces its virulence, and animal tests using one of the recombinant viruses show great lethality and transmissibility in pigs. The isothermal technique known as recombinase polymerase amplification (RPA) is perfect for rapid in-field detection. To accurately identify ASFV MGF-505R gene-deleted mutants and assess the complex infection situation of ASF, RPA assays in conjunction with real-time fluorescent detection (real-time RPA assay) and lateral flow dipstick (RPA-LFD assay) were created. These innovative methods allow for the direct detection of ASFV from pigs, offering in-field pathogen detection, timely disease management, and satisfying animal quarantine requirements. The specific primers and probes were designed against conserved regions of ASFV B646L and MGF-505R genes. Using recombinant plasmid DNA containing ASFV MGF-505R gene-deleted mutants as a template, the sensitivity of both ASF real-time RPA and ASF RPA-LFD assays were demonstrated to be 10 copies per reaction within 20 min at 37 °C. Neither assay had cross-reactions with CSFV, PRRSV, PPV, PRV, ot PCV2, common viruses seen in pigs, indicating that these methods were highly specific for ASFV. The evaluation of the performance of ASFV real-time RPA and ASFV RPA-LFD assays with clinical samples (*n* = 453) demonstrated their ability to specifically detect ASFV or MGF-505R gene-deleted mutants in samples of pig feces, ham, fresh pork, and blood. Both assays exhibited the same diagnostic rate as the WOAH-recommended real-time fluorescence PCR, highlighting their reliability and validity. These assays offer a simple, cost-effective, rapid, and sensitive method for on-site identification of ASFV MGF-505R gene-deleted mutants. As a promising alternative to real-time PCR, they have the potential to significantly enhance the prevention and control of ASF in field settings.

## Simple Summary

African swine fever (ASF) is a devastating infectious disease in pigs, necessitating rapid and accurate disease diagnosis. In this study, to accurately identify ASFV MGF-505R gene-deleted mutants and assess the complex infection situation of ASF, recombinase polymerase amplification (RPA) assays coupled with real-time fluorescent detection (real-time RPA assay) and lateral flow dipstick (RPA-LFD assay) were developed for on-site detection. These methods, targeting conserved regions of ASFV B646L and MGF-505R genes, demonstrated high sensitivity (10 copies/reaction in 20 min at 37 °C) and specificity, with no cross-reactivity to other common pig viruses. Clinical testing (*n* = 453) confirmed their reliability, matching the diagnostic rate of WOAH-recommended real-time PCR. These assays provide a simple, cost-effective, and rapid solution for field detection, enhancing ASF prevention and control.

## 1. Introduction

African swine fever (ASF), a devastating disease of swine, was first reported in Kenya in the 1920s [1] and has since spread to Europe, Western Europe, and then to South America and the Caribbean [2]. It was eventually eradicated by the mid-1990s, except in Sardinia [3]. However, ASF has spread into Eastern Europe and Russia since its second major outbreak, initially into Georgia in 2007 [4,5]. African swine fever virus (ASFV) has continued to spread around the world, including in China [6]. In 2018, the Georgia-07-like genotype II ASFV emerged in China, and has been prevalent in China for almost three years. Two non-hemadsorbing genotype I ASFVs, HeN/ZZ-P1/21 and SD/DY-I/21, were isolated from pig farms in Henan and Shandong province, respectively, and both isolates showed a deletion of the MGF-505R gene [7]. Thereafter, three recombinants of genotype I and II ASFVs in pigs, JS/LG/2, HeN/123014/22, and IM/DQDM/22, were detected in China [8]. Although these recombinants share genetic similarities and are categorized as genotype I based on their B646L gene, 10 distinct fragments that make up more than 56% of their genomes are obtained from viruses of genotype II. Animal research revealed that the virulence of a virulent genotype II virus is significantly reduced when the virulence-related genes MGF_505/360 and EP402R are deleted. These above studies showed that the deletion of the MGF-505R gene may be an important sign for the newly emerging recombinant ASFVs, and a rapid detection method targeting MGF-505R should be established.

Several molecular tools (for example, polymerase chain reaction (PCR), real-time PCR, ELISA, and viral isolation) have provided crucial support and genetic information for the study of ASFV [9,10,11]. However, they are not suitable for field (on-site) detection in pig farms due to factors such as equipment requirements, time constraints, and sample processing challenges. Recently, recombinase polymerase amplification (RPA) has emerged as a promising alternative to PCR as a new method of DNA amplification [12]. With its isothermal nature and rapid amplification kinetics, RPA offers a versatile and efficient tool for diagnostic applications. Compared to other commonly used molecular diagnostic methods, RPA reactions can be finished in about 20 min and are performed at a constant temperature, saving the need for an expensive thermocycler [13]. RPA is suitable for on-site field testing of clinical samples [14,15].

One isothermal technique for amplifying DNA/RNA is recombinase polymerase amplification (RPA) that relies on three enzymes: a recombinase, a single-stranded DNA binding protein (SSB), and a polymerase [12]. The recombinase enzyme is essential for matching oligonucleotide primers with homologous sequences in the target DNA during the RPA reaction. This pairing process is essential for the subsequent steps of the reaction. Once the primers have annealed to the target DNA, the SSB binds to the displaced strand, stabilizing the primer–template complex and preventing primer dissociation. Finally, the polymerase enzyme initiates DNA synthesis by adding nucleotides to the 3′ end of the primers. This process is rapid, allowing for the amplification of even very low concentrations of target DNA. RPA can amplify as little as 1–10 DNA target copies within 10 to 20 min at constant temperatures ranging from 37–42 °C.

In this study, recombinase polymerase amplification (RPA) assays in conjunction with lateral flow dipstick (RPA-LFD assay) and real-time fluorescent detection (real-time RPA assay) were successfully established for rapid and specific detection of ASFV MGF-505R gene-deleted mutants.

## 2. Materials and Methods

### 2.1. Ethics Statement

This study was approved by the Ethics Committee of the Institute of Animal Quarantine, Chinese Academy of Inspection and Quarantine. Clinical samples (pig blood, feces, fresh pork, viscera, and pig ham) from pig farms were obtained with the approval of the farm manager. Pigs were treated in accordance with the Animal Ethics Procedures and Guidelines of the People’s Republic of China during the specimen collection process.

### 2.2. Virus Strains, Reference DNA, and Clinical Samples

Classical swine fever virus (CSFV, Chinese Hog Cholera Lapinised Virus C strain, CVCC AV1535), respiratory and reproductive syndrome virus (PRRSV, JXA1-R strain, Veterinary Drug Production License No.101041064), pseudorabies virus (PRV, EA strain, Veterinary Drug Production License No.220051044), porcine circovirus-2(PCV-2, ZJ/C strain, Veterinary Drug Production License No. 221011188), and porcine parvovirus (PPV, WH-1 strain, Veterinary Drug Production License No. 220051059) were all from the commercial attenuated live vaccine. A total of 453 clinical samples (105 pig blood, 9 pig feces, 178 fresh pork, 39 cooked pork, 7 cooked pig viscera, 8 fresh pig viscera, and 107 pig ham) were collected from pig farms/supermarkets between the years 2019 and 2024. Viral DNA/RNA was extracted utilizing the TIANamp Virus DNA/RNA kit (Tiangen Biotech Co., Ltd., Beijing, China) in accordance with the manufacturer’s guidelines. All RNA and DNA templates were stored at −80 °C and preserved in our laboratory for further analysis.

The ASFV B646L gene and MGF-505R gene were synthesized by BGI Genomics Co., Ltd. (Beijing, China) based on the reference sequence of ASFV (GenBank: MK333180.1), and cloned into the plasmid vector pUC57. The ASFV MGF-505R gene-deleted mutants were respectively constructed by using the Genbank database (MZ945537.1: 21034-19686 and MZ945536.1: 20336-18988) of Pig/SD/DY-I/2021 and Pig/HeN/ZZ-P1/2021. Those recombinant plasmids were amplified in Escherichia coli DH5a cells, extracted, and purified using the TaKaRa MiniBEST Plasmid Purification Kit. Plasmid DNA was quantified using a Nanodrop ONE spectrophotometer, and then it was stored at −80 °C before RPA analysis.

### 2.3. Design of Primers and Probes

In the present study, RPA methods were employed to efficiently detect ASFV, specifically targeting the highly conserved B646L and MGF-505R1 sequences (GenBank: MK333180.1).

The amplification product was fewer than 500 bp, the amplification efficiency was good, and the RPA primer was 30–35 nt long. With 30 and 15 nucleotides positioned at 5′ and 3′, respectively, to an internal base analog tetrahydrofuran (THF), the Exo probe was a long oligonucleotide (46–52 bases). The reverse primer for RPA-LFD added a biotin on the 5′ end. RPA-LFD probe was added a FAM on the 5′ end, a C3 spacer on the 3′ end, a dSpacer (tetrahydrofuran, THF) in the middle.

Based on the reference sequence of the conserved region of the ASFV B646L and MGF-505R gene, many primers and probes were created in accordance with the RPA primer principles (Table 1). Firstly, the exo and nfo probes targeting B646L/MGF505R were designed according to the probe design considerations described forwardly. With the aim of amplifying sequences adjacent to the target probes, a total of six kinds of primer combinations targeting B646L (B646L-RPA-F/B646L-RPA-R, B646L-RPA-F1/646L-RPA-R1, B646L-RPA-F2/B646L-RPA-R2, B646L-LFA-F/B646L-LFA-R, B646L-LFA-F1/B646L-LFA-R1, B646L-LFA-F2/B646L-LFA-R2) and six kinds of primer combinations targeting MGF-505R (MGF505R-RPA-F/MGF505R-RPA-R, MGF505R-RPA-F/MGF505R-RPA-R2, MGF505R-RPA-F1/MGF505R-RPA-R1, MGF505R-LFA-F/MGF505R-LFA-R, MGF505R-LFA-F1/MGF505R-LFA-R, MGF505R-LFA-F2/MGF505R-LFA-R2) were designed for the RPA assays (Table 1).

All the primers and probes were synthesized by BGI Genomics Co., Ltd. (Beijing, China).

### 2.4. Screening the Primer Recombinations for ASFV Real-Time RPA and RPA-LFD Assays

The standard basic RPA assays were performed in a 50 µL reaction volume utilizing the DNA Isothermal Rapid Amplification Kit (Basic) procured from Amplification Future Biotechnology Co., Ltd. (Changzhou, China). In order to create the master mix, 12 µL of nuclease-free H_2_O and 29.4 µL of rehydration buffer A were combined with 2 µL of RPA primers (forward/reverse primer pairs, each 10 µM). The lyophilized RPA enzyme powder was added to a 0.2 mL reaction tube along with the master mix. The reaction tube was then pipetted with 2 µL of template DNA. Parallel treatment was given to the internal and blank control templates. Lastly, each tube received 2.5 µL of buffer B, which was 280 mM magnesium acetate. The tube was immediately put in a portable isothermal nucleic acid detection device (Isothermal Fluorescence Detector WL-16-III; Amplification Future, Changzhou, China) after the basic RPA reactions had been gently mixed and quickly vortexed. Then, the RPA products were analyzed with 3% agarose gel electrophoresis.

### 2.5. Reaction Systems and Reaction Conditions for ASFV Real-Time RPA and RPA-LFD Assays

The DNA Isothermal Rapid Amplification Kit (Fluorescent Type) from Amplification Future (Changzhou) Biotechnology Co., Ltd., Changzhou, China, was used to conduct the RPA reactions in a 50 µL reaction volume. The real-time RPA reaction components included 2 µL of RPA primers (forward/reverse primer pairs, each 10 µM), 0.6 µL of exo probes (probe, 10 µM), and 11.4 µL of nuclease-free H_2_O was mixed with 29.4 µL of rehydration buffer A to make the master mix. The master mix was dispensed into 0.2 mL reaction tubes, each containing a precise amount of lyophilized RPA enzyme powder. Subsequently, 2 µL of template DNA was added to the reaction tube via pipetting. Parallel treatment was given to the internal and blank control templates. Lastly, each tube received 2.5 µL of buffer B, which was 280 mM magnesium acetate. The tube was immediately put in a portable isothermal nucleic acid detection device (Isothermal Fluorescence Detector WL-16-III; Amplification Future, Changzhou, China) after the real-time RPA reactions had been gently mixed and quickly vortexed. The fluorescence signal, which rose significantly with successful amplification, was recorded in real time at 39 °C using the FAM channel every 30 s for 20 min (end-point reading).

The RPA-LFD reactions were performed in a 50 µL reaction volume utilizing the DNA Isothermal Rapid Amplification Kit (Colloidal Gold Test Strip Type) procured from Amplification Future Biotechnology Co., Ltd. (Changzhou, China). The RPA-LFD reaction components included 2 µL of RPA primers (forward/reverse primer pairs, each 10 µM), 0.6 µL of RPA-LFD probes (probe, 10 µM), 11.4 µL of nuclease-free H_2_O, 29.4 µL of rehydration buffer A, and 2 µL of template DNA, and each tube was then filled with 2.5 µL of 280 mM magnesium acetate (Buffer B). The tube was immediately put in the thermostat water bath at different temperatures (37 °C, 39 °C, and 41 °C) for 15 min after the RPA-LFD reactions had been gently mixed and briefly vortexed. In order to enable the specific detection of FAM-labeled amplicons that are visible to the unaided eye in less than five min, this ASFV RPA-LFD assay was then coupled with a portable lateral flow device (Amplification Future (Changzhou) Biotechnology Co., Ltd., Changzhou, China) that had two distinct detection lines coupled with one positive control line.

### 2.6. Sensitivity and Specificity of ASFV Real-Time RPA and RPA-LFD Assays

To evaluate the sensitivity, pUC-MGF-505R and pUC-B646L recombinant plasmid were serially diluted 10-fold to achieve concentrations ranging from 10^4^ to 10^1^ copies per reaction and were amplified by both real-time RPA and RPA-LFD assays. To determine the specificity, 10^6^ copies of each template (i.e., nucleic acid of CSFV, PRRSV, PPV, PRV, PCV2, and Mut-MGF-505R-He21/SD21) were amplified by real-time RPA and RPA-LFD reactions. Considering the 3Rs method, recombinant plasmids were utilized for experiments [16].

### 2.7. ASFV Real-Time RPA and RPA-LFD Assays on Clinical Samples

To further evaluate the performance of ASFV real-time RPA and RPA-LFD methods, clinical samples (*n* = 453), including pig blood, ham, pork (fresh or cooked), pig viscera (fresh or cooked), and feces, were detected, and the detection results were compared with WOAH-recommended real-time fluorescence PCR method [10].

## 3. Results

### 3.1. Primers Design and Screening

**Figure 1:**
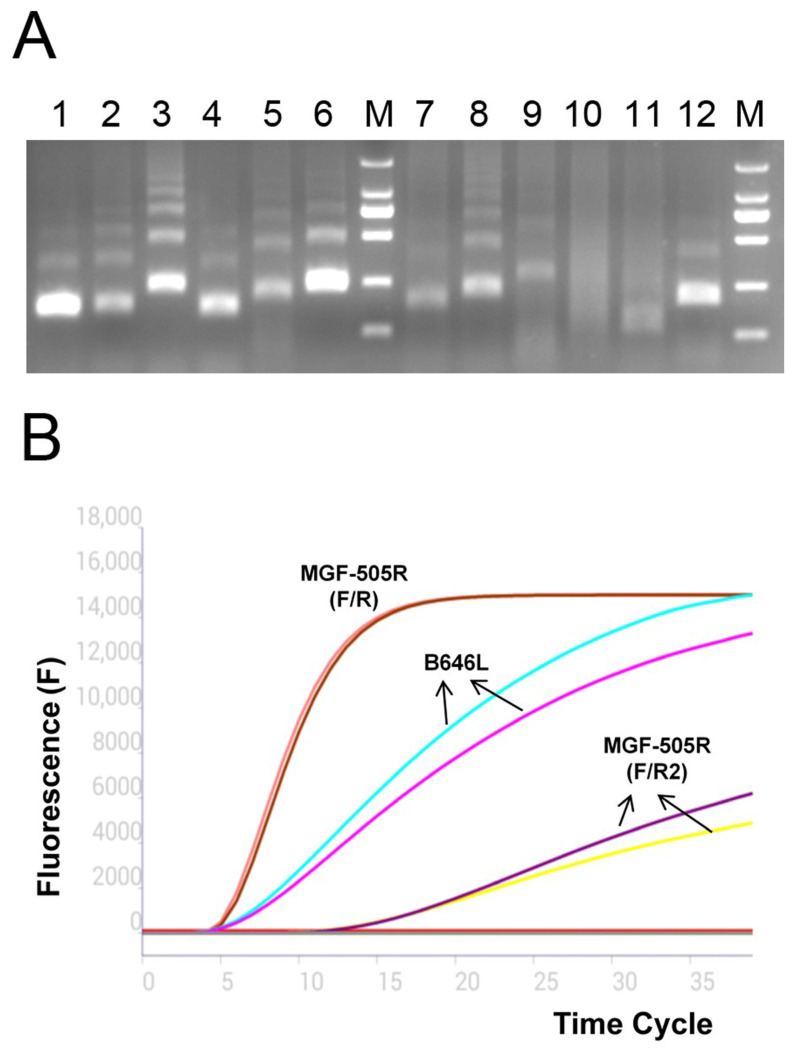
The screening for the optimal primers and probe combination for ASFV dual real-time RPA methods targeting (**A**) MGF-505R and (**B**) B646L.

The ASFV B646L gene encodes the viral capsid protein P72 that plays an essential role in virion assembly and virus attachment to the host cell [17,18]. ASFVs are divided into 24 genotypes based on the C-terminal sequence of their B646L gene with 86.2–99.5% nucleotide identity [19]. The sequences of screened reference strains of the African swine fever virus, utilized in the design of primers and probes, are detailed in Table 2. In the primer screening assay, the primer combinations B646L-RPA-F/B646L-RPA-R, B646L-RPA-F1/646L-RPA-R1, B646L-RPA-F2/B646L-RPA-R2, B646L-LFA-F/B646L-LFA-R, B646L-LFA-F1/B646L-LFA-R1, B646L-LFA-F2/B646L-LFA-R2, MGF505R-RPA-F/MGF505R-RPA-R, MGF505R-RPA-F/MGF505R-RPA-R2, and MGF505R-LFA-F2/MGF505R-LFA-R2 yielded the obvious products on the agarose gel (Figure 1A). Considering the amplification efficiency and the size of the products, B646L-RPA-F/B646L-RPA-R, B646L-LFA-F/B646L-LFA-R, MGF505R-LFA-F2/MGF505R-LFA-R2, MGF505R-RPA-F/MGF505R-RPA-R, and MGF505R-RPA-F/MGF505R-RPA-R2 were selected for further examination.

The screened RPA primer combinations were tested with pUC-MGF-505R and pUC-B646L recombinant plasmids in real-time RPA. The results showed that MGF-505R-RPA-F combined with MGF-505R-RPA-R had a good effect, but the amplification efficiency was poor when combined with MGF-505R-RPA-R2. In addition, B646L-RPA-F and B646L-RPA-R were also highly amplified. Therefore, the targeted primer pairs MGF-505R-RPA-F/R and B646L-RPA-F/R were chosen for further verification, indicating that they generated the first and highest fluorescence signals (Figure 1B).

**Figure 2:**
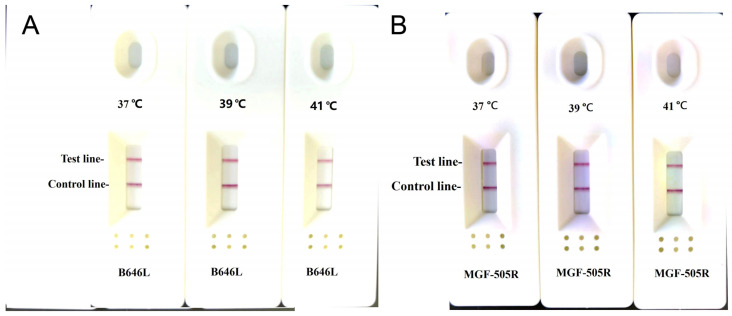
Screening the optimal reaction temperatures for RPA-LFD assays (**A**) B646L and (**B**) MGF-505R.

The screened LFA primer combinations were tested with pUC-MGF-505R and pUC-B646L recombinant plasmids in RPA-LFA. The reaction mixture was incubated at three different temperatures (37 °C, 39 °C, and 41 °C) for 15 min in order to identify the ideal reaction temperature for the RPA-LFD assay. The amplicons were monitored using a portable fluorescence detector. The ideal temperature for RPA-LFD reactions was discovered to be 39 °C. The amplification response was not significantly affected by higher incubation temperatures (Figure 2).

### 3.2. Sensitivity and Specificity of Real-Time RPA and RPA-LFD Assays

**Figure 3:**
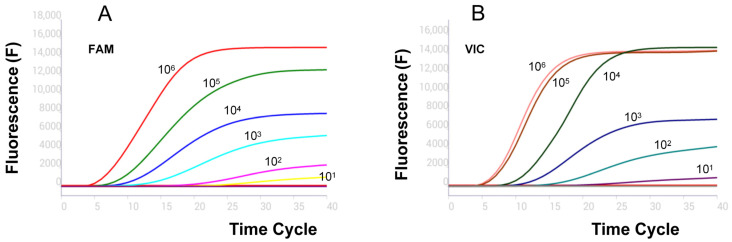
Sensitivity test of dual real-time RPA assays (**A**) B646L and (**B**) MGF-505R.

**Figure 4:**
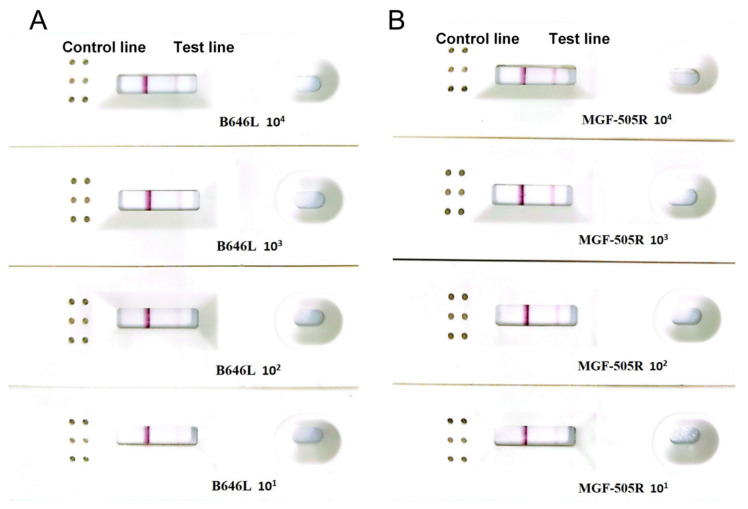
Sensitivity test of RPA-LFD assays (**A**) B646L and (**B**) MGF-505R.

To determine the analytical sensitivity, a tenfold dilution range of the recombinant plasmid (2.5 × 10^−1^~2.5 × 10^5^ copies/µL corresponds to 10^6^~10^1^ copies per reaction, Figure 3) was used to evaluate the RPA-LFD and real-time RPA assays. The results showed that they were sufficiently sensitive to detect 10^1^ copies per reaction (Figure 4).

**Figure 5:**
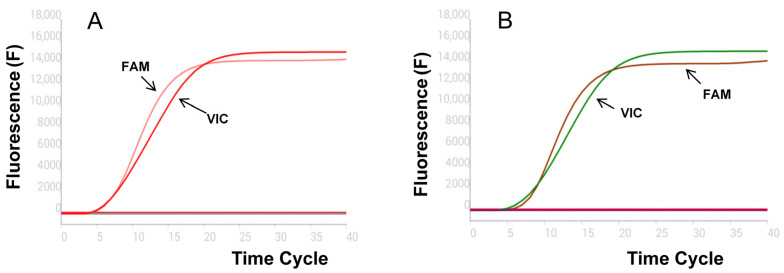
Specificity results of real-time RPA assays (**A**) B646L and (**B**) MGF-505R.

The specificity tests were conducted using the nucleic acids of CSFV, PRRSV, PPV, PRV, and PCV2. The results showed that only positive controls (pUC-MGF-505R and pUC-B646L were used as templates) could show specific fluorescence curves, while the rest were all negative (Figure 5A). This proves that the established dual fluorescence RPA method can simultaneously detect the B646L and MGF-505R genes of ASFV, with no cross-reaction with other pathogen nucleic acids, exhibiting good specificity. Furthermore, the specificity of the dual fluorescence RPA method was further verified using Mut-MGF-505R-He21/SD21. No amplification curve was observed (Figure 5B).

**Figure 6:**
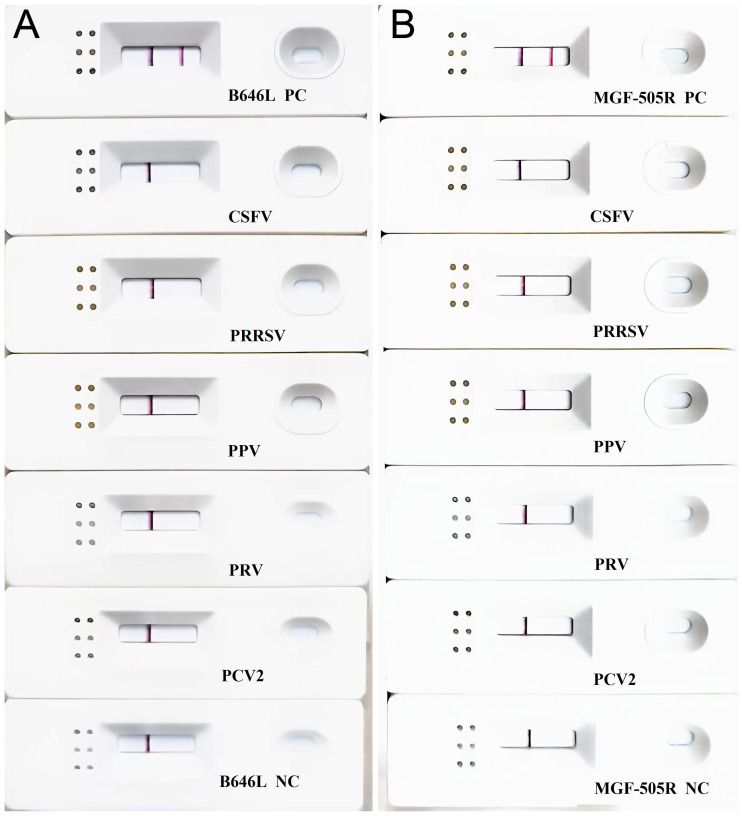
Specificity results of RPA-LFD assays (**A**) B646L and (**B**) MGF-505R.

Regarding the specificity of RPA-LFA, there were no identified cross-reactions with other significant pig viruses (Figure 6).

All the experiments were repeated three times with similar results.

### 3.3. Repetitiveness Test for Real-Time RPA

The recombinant plasmids of pUC-MG505R and pUC-B646L at different dilution concentrations were used as templates. The optimized fluorescent RPA method was employed for repeatability testing. The results showed that the coefficients of variation within each group for FAM and VIC channels ranged between 0.4% and 0.65%, all less than 2%. This indicates that the established dual fluorescent RPA method has good repeatability.

### 3.4. Performance of Real-Time RPA and RPA-LFD Assay on Clinical Samples

Of the 453 samples, 191 samples were positive for B646L and MGF-505R, and the remaining samples were negative in the above-mentioned three methods (Table 3). With this data, the clinical specificity of real-time RPA and RPA-LFD assays could be calculated as 100%. The real-time RPA and RPA-LFD tests were faster than real-time PCR, despite the fact that the detection outcomes from the three techniques were consistent. Specifically, real-time PCR produced findings around two h later than the whole RPA experiment, which produced data in 30 min.

(A) Agarose gel electrophoresis of RPA products was generated by twelve sets of primer combinations based on B646L and MGF-505R gene sequences: 1, B646L-RPA-F/B646L-RPA-R (166 bp); 2, B646L-RPA-F1/646L-RPA-R1 (165 bp); 3, B646L-RPA-F2/B646L-RPA-R2 (229 bp); 4, B646L-LFA-F/B646L-LFA-R (160 bp); 5, B646L-LFA-F1/B646L-LFA-R1 (205 bp); 6, B646L-LFA-F2/B646L-LFA-R2 (236 bp); 7, MGF505R-RPA-F/MGF505R-RPA-R (173 bp); 8, MGF505R-RPA-F/MGF505R-RPA-R2 (207 bp); 9, MGF505R-RPA-F1/MGF505R-RPA-R1 (267 bp); 10, MGF505R-LFA-F/MGF505R-LFA-R (142 bp); 11, MGF505R-LFA-F1/MGF505R-LFA-R (115 bp); 12, MGF505R-LFA-F2/MGF505R-LFA-R2 (194 bp); M, DL2000 marker. (B) The screened RPA primer combinations were tested in real-time RPA. MGF-505R (F/R) means the combination of MGF-505R-RPA-F combined with MGF-505R-RPA-R while the MGF-505R (F/R2) means the combination of MGF505R-RPA-F/MGF505R-RPA-R2.

## 4. Discussion

More than 100 non-structural proteins and 68 structural proteins can be encoded by ASFV [20]. The B646L gene encodes the p72 protein, which is found on the viral capsid surface and has the capacity to both actively participate in the viral attachment process to host cells and trigger the host’s generation of neutralizing antibodies. [21]. ASFVs are classified into different genotypes based on the 3′-end sequences of the B646L gene, which encodes the major capsid protein p72 [10,11]. ASFVs can be classified into 24 genotypes, with a nucleotide identity of 86.2 to 99.5%, according to their B646L gene, which reflects the genetic diversity of ASFV [19]. This diversity can affect the virulence, host range, and antigenic properties of different ASFV strains. However, the high conservation of the P72 gene among these genotypes makes it a suitable target for the development of broad-spectrum ASFV detection methods. In this research, the B646L gene was identified as the conserved target for ASFV detection, consistent with numerous prior studies [22,23,24].

The multigene family (MGF) is located at both ends of African swine fever virus genome and is the main cause of changes in genome length for different ASFVs [25]. The families are named based on the average number of amino acids encoding the proteins and within each MGF, and the genes are named according to the direction of gene reading and their position within that family [26]. The families include MGF100, MGF110, MGF300, MGF360, and MGF505/MGF530. African swine fever virus (ASFV) isolate BA71V contains seven homologous genes, with an average of 505 amino acids encoding, so it was given the name multigene family 505 (MGF505) [27]. The ASFV MGF-505R gene is a latent virulence gene and immune escape gene, which can decrease the virulence and enhance the host immunity [20,28,29]. In addition, it can serve as a potential target gene for vaccine development. Although the ASFV MGF-505R gene was deleted alone or combined with the CD2V gene in several previous studies for vaccine development, it has not been approved for mass production and use due to safety and protection [30]. As a result, there is currently no effective treatment or vaccine available for ASFV, and disease control primarily relies on the slaughter of infected animals and strict sanitary measures. Given the rapid spread of ASF and its high mortality rate, timely identification and confirmation of the disease are crucial for effective control and prevention. Therefore, this study established a rapid detection method for ASFV MGF-505R gene-deleted mutants by using the ASFV MGF-505R gene as a target and combining it with the conserved B646L gene, and these were further compared with WOAH-recommended real-time fluorescence PCR for the detection of field samples. Ultimately, we established a promising, cost-effective, highly sensitive, and selective method for on-site detection of ASFV MGF-505R gene-deleted mutants suitable for pig farms.

The isothermal gene amplification-based assay has surfaced as an intriguing alternative to PCR for diagnosing diseases at the molecular level. Until now, some types of isothermal amplification techniques have been explored for the detection of ASFV: cross-priming amplification (CPA) [31], ladder-shape melting temperature isothermal amplification (LMTIA) [32], and Loop-Mediated Isothermal Amplification (LAMP) [33]. CPA was developed by Frączyk et al. [31] for fast and direct detection of ASFV in the blood and sera of infected pigs and wild boars, and the detection limit was 7.2 copies of a diluted, standard ASFV plasmid. Wang et al. [32] developed an LMTIA assay, which can detect 10^4^ dilutions of DNA extracted from the positive reference serum sample to detect ASFV. Wang et al. [33] developed the LAMP assay to detect ASFV with a detection limit of 30 copies/μL of pUC57 containing the p10 gene sequence, which is slightly less sensitive than our real-time RPA and RPA-LFD assay. Compared with RPA, LAMP uses six primers, whose design and selection are very challenging, and primer–primer interaction in LAMP reactions can result in false positive signals.

Although RPA has been extensively studied for the detection of various pathogens such as ASFV, its application in combination with real-time fluorescent detection (real-time RPA assay) and lateral flow dipstick (RPA-LFD assay) for the detection of MGF-505R gene-deleted mutants of African swine fever virus (ASFV) has not been forwardly reported. Several RPA/RAA methods have been developed for on-site detection of ASFV, with commonly utilized target sequences including B646L, CD2V, and EP402R genes [34,35,36]. The innovative approach developed in this study, incorporating real-time RPA and RPA-LFD assays, holds considerable promise for improving the accuracy and speed of ASFV mutant detection. The method has a high clinical application value and a wide promotion prospect, which provides new technical support for the rapid on-site detection of ASFV for the subsequent healthy development of China’s pig breeding industry.

ASFV B646L, MGF505-7R gene, E183L gene, 9GL gene, E248R gene, and A137R gene are among the qPCR techniques developed for laboratory testing that have different limits of detection (LOD) ranging from 2.63 to 20 copies/μL [37,38,39,40]. Meanwhile, several detection methods for on-site testing, such as RPA, LAMP, CPA, and CRISPR, have been devised with LODs ranging from 1 to 5.7 × 10^4^ copies/μL [34,35,41,42,43,44]. In this study, the RPA primers were designed on the B646L MGF-505R gene of ASFV as a target, and the real-time RPA and RPA-LFD assays for the detection of ASFV were established. As shown by the experiments, the technique for real-time RPA and RPA-LFD is reproducible. When the plasmid containing the target sequence was used as a template, the detection limit of the real-time RPA and RPA-LFD assays was 2.5 copies/μL pUC-MGF-505 and pUC-B646L DNA, which is equivalent to 10 copies per reaction. The established double detection methods employed for on-site utilization demonstrate good sensitivity when compared to their predecessors. Besides, these detection methods do not rely on large-scale equipment facilities in the laboratory and could swiftly execute gene typing of ASFV in the field.

The specificity of the real-time RPA and RPA-LFD assays was determined with several virus strains (i.e., CSFV, PRRSV, PPV, PRV, PCV2), as in traditional practice, and the real-time RPA and RPA-LFD assays were also compared with the WOAH-recommended real-time fluorescence PCR. The test results of 453 clinical samples indicated that the RPA and RPA-LFD assays had the same diagnostic rate as the WOAH-recommended real-time fluorescence PCR of 100%, highlighting their reliability and validity. However, the real-time RPA and RPA-LFD assays developed in this study were only validated with clinical African swine fever virus (ASFV) samples but not the ASFV MGF-505R gene-deleted mutants, which is a limit of this study. Further validation with more ASFV and gene-deleted mutant samples is needed for the real-time RPA and RPA-LFD assays.

## 5. Conclusions

In this study, two RPA-based assays (real-time RPA and RPA-LFD) were developed for on-site detection of ASFV and MGF-505R gene-deleted mutants. These methods, targeting conserved regions of ASFV B646L and MGF-505R genes, demonstrated high sensitivity (10 copies/reaction in 20 min at 37 °C) and specificity, with no cross-reactivity to other common pig viruses. Clinical testing (*n* = 453) confirmed their reliability, matching the diagnostic rate of the WOAH-recommended real-time PCR. These assays provide a simple, cost-effective, and rapid solution for field detection, enhancing ASF prevention and control.

## Figures and Tables

**Table 1:** Primers and probes used in ASFV real-time RPA and ASFV RPA-LFD assays.

Name	Sequence (5′–3′)	Location (MK333180.1)
MGF505R-RPA-F	CATGTAAAGATCATAATTATGAAGTTATTAA	28255-28285
MGF505R-RPA-R	ATCTAAATGATGATACTTATCGGGTACGATTC	28396-28427
MGF505R-RPA-R2	TATGTAGGAGTTGTAGGCTTGAAAGCATGCG	28431-28461
MGF505R-RPA-F1	CACTCATTTACTTATTGAAAAGGCATGTAA	28232-28261
MGF505R-RPA-R1	AGGATAAAATCTAAGTATCCTTTGGCTGCCA	28465-28495
MGF505R-RPA-P	ATGTGCTATTGCCCATAAGGATCTACATCTA/i6FAMdT/A/idSp//iBHQ1dT/GTTTGGGGTATAGA/iSpC3/	28334-28382
MGF505R-LFA-F	GAAAACCTACATATCTACAATATGATAGATACC	28296-28328
MGF505R-LFA-F1	AGATACCTTTGAATGTGCTATTGCCCATAAGG	28322-28353
MGF505R-LFA-R	5′Biotin-CATGCGAATATCTAAATGATGATACTTATCGGG	28404-28436
MGF505R-LFA-F2	AATGGATATATGAAAACCTACATATCTAC	28285-28313
MGF505R-LFA-R2	5′Biotin-TCCTTTGGCTGCCACCTTATGTAGGAGTTGT	28448-28478
MGF505R-LFA-P	5′FAM-ATGTGCTATTGCCCATAAGGATCTACATCTATA/idSp/TGTTTGGGGTATAGA/iSpC3/	28334-28382
B646L-RPA-F	TGAAAGCTTATCTCTGCGTGGTGAGTGGGCTGC	104033-104065
B646L-RPA-R	AACTAATGTCTGCTCTTAAATGGCCCATTGA	104168-104198
B646L-RPA-F1	TATCCTGAAAGCTTATCTCTGCGTGGTGAGTGG	104028-104060
B646L-RPA-R1	TGTCTGCTCTTAAATGGCCCATTGAATATATG	104161-104192
B646L-RPA-F2	GCGTCTGGAAGAGCTGTATCTCTATCCTGAAAGC	104006-104039
B646L-RPA-R2	AGGTGACCCACACCAACAATAACCACCACGATG	104202-104234
B646L-RPA-P	TGGCGTTAACAACATGTCCGAACTTGTGCCAA/iVICdT/T/idSp//iBHQ1dT/CGGTGTTGATGAGGA/iSpC3	104070-104119
B646L-LFA-F	TGAGGATTTTGATCGGAGATGTTCCAGGTAGGT	104114-104146
B646L-LFA-R	5′Biotin-AACGCGTTCGCTTTTCGCTGATACGTGTCCAT	104242-104273
B646L-LFA-F1	TGTTGATGAGGATTTTGATCGGAGATGTTCCA	104108-104139
B646L-LFA-R1	5′Biotin-ACCTGTTTGTAACCCCTGAAATACACAACCT	104282-104312
B646L-LFA-F2	GAACTTGTGCCAATCTCGGTGTTGATGAGGA	104089-104119
B646L-LFA-R2	5′Biotin-TTTACATCAATAACCTGTTTGTAACCCCTGA	104294-104324
B646L-LFA-P	5′FAM-AGCAGACATTAGTTTTTCATCGTGGTGGTTATT/idSp/TTGGTGTGGGTCACCT/iSpC3/	104185-104234

**Table 2:** The sequences of screened reference strains of the African swine fever virus utilized in the design of primers and probes.

Serial Number	GenBank ACCESSION Number	Virus Strains	Genotype	Isolation Location/Host
1	AF449461	Madrid/62	I	Spain/domestic pig
2	AF301543	Malta/78	I	Malta/wild boar Sus scrofa
3	AF301537	Lisbon/57	I	Portugal/wild boar Sus scrofa
4	FJ174367	Ori93	I	Italy/domestic pig
5	AM999764	Georgia 2007	II	Georgia/domestic pig
6	MH713612	CN-SY18	II	China/domestic pig
7	MK189456	JILIN2018	II	China/wild boar Sus scrofa
8	JF260952	Rostov-08-10	II	Russia/domestic pig
9	KJ627217	Pol14/Sz	II	Poland/European wild boar
10	AY351517	MOZ/2002/1	II	Mozambique/wild boar Sus scrofa
11	AF504886	BOT/1/99	Ⅲ	Botswana/domestic pig
12	JX294722	RSA 2011/01	Ⅲ	South Africa/domestic pig
13	DQ250124	RSA/5/95	IV	South Africa/domestic pig
14	JX467630	RSA 99.1	IV	South Africa/domestic pig
15	AF301541	Tengani	V	Malawi/wsarthog
16	AF270711	MOZ/94/1	VI	Mozambique/domestic pig
17	AF302818	RSA/1/9	VII	South Africa/domestic pig
18	AY274457	MOZ-62/98	VⅢ	Mozambique/domestic pig
19	AF270707	Malawi/1978	VⅢ	Malawi/*
20	HQ645943	Con09/Ni16	IX	Congo/domestic pig
21	AF449475	UGA/1/1995	IX	Kenya/domestic pig
22	AF449463	BUR 1/1984	X	Burundi/domestic pigs
23	AY351522	KAB/62	XI	Zambia/tick
24	AY351543	MZI/1/92	XII	Malawi/wild boar
25	AY351542	SUM/1411	XⅢ	Zambia/tick
26	AY351555	NYA/12	XIV	Zambia/tick
27	AY494552	TAN/1/01	XV	Tanzania/domestic pig
28	AY494551	TAN/2003/2	XVI	Tanzania/domestic pig
29	DQ250119	ZIM/92/1	XVII	Zimbabwe/domestic pig
30	DQ250122	NAM/1/95	XVⅢ	Namibia/domestic pig
31	DQ250127	RSA/3/96	XIX	South Africa/domestic pig
32	DQ250109	lillie	XX	South Africa/domestic pig
33	DQ250125	RSA/1/96	XXI	South Africa/domestic pig
34	DQ250117	SPEC/245	XXII	South Africa/domestic pig
35	KT795360	ETH/3	XXⅢ	Ethiopia/wild boar Sus scrofa
36	KY353989	MOZ/10/2006	XXIV	Mozambique/soft tick

* means that the information cannot be determined.

**Table 3:** Comparison of real-time RPA and RPA-LFD assays with real-time qPCR assay on clinical samples.

Clinical Sample Type	Real-Time PCR (qPCR)		Real-Time RPA/RPA-LFD Assays	
	MGF-505R (Positive/Total Samples)	B646L (Positive/Total Samples)	MGF-505R (Positive/Total Samples)	B646L (Positive/Total Samples)
Pig feces	1/9	1/9	1/9	1/9
Pig hams	74/107	74/107	74/107	74/107
Fresh pork	81/178	81/178	81/178	81/178
Pig blood	10/105	10/105	10/105	10/105
Cooked pig viscera	2/7	2/7	2/7	2/7
Pig viscera	5/8	5/8	5/8	5/8
Cooked pork	18/39	18/39	18/39	18/39
Total	191/453	191/453	191/453	191/453

## Data Availability

Data are contained within the article.

## Abbreviations

The following abbreviations are used in this manuscript:

ASF	African swine fever
ASFV	African swine fever virus
LFD	Lateral flow dipstick
RPA	Recombinase polymerase amplification
WOAH	World Organisation for Animal Health
CPA	Cross-priming amplification
LAMP	Loop-Mediated Isothermal Amplification
LMTIA	Ladder-shape melting temperature isothermal amplification
LOD	Limits of detection
